# Effects of Tai Chi Chuan on Cognitive Function in Adults 60 Years or Older With Type 2 Diabetes and Mild Cognitive Impairment in China: A Randomized Clinical Trial

**DOI:** 10.1001/jamanetworkopen.2023.7004

**Published:** 2023-04-06

**Authors:** Yannan Chen, Jiawei Qin, Liyuan Tao, Zhizhen Liu, Jia Huang, Weilin Liu, Ying Xu, Qiang Tang, Yongguo Liu, Zhuhong Chen, Shangjie Chen, Shengxiang Liang, Cong Chen, Jinjin Xie, Jue Liu, Lidian Chen, Jing Tao

**Affiliations:** 1 College of Rehabilitation Medicine, Fujian University of Traditional Chinese Medicine, Fuzhou, China; 2 Department of Rehabilitation Medicine, Quanzhou First Hospital, Fujian Medical University, Quanzhou, China; 3 Research Center of Clinical Epidemiology, Peking University Third Hospital, Beijing, China; 4 National-Local Joint Engineering Research Center of Rehabilitation Medicine Technology, Fujian University of Traditional Chinese Medicine, Fuzhou, China; 5 Second Affiliated Hospital of Heilongjiang University of Traditional Chinese Medicine, Haerbin, China; 6 Knowledge and Data Engineering Laboratory of Chinese Medicine, School of Information and Software Engineering, University of Electronic Science and Technology of China, Chengdu, China; 7 Xiyuan Hospital, China Academy of Chinese Medical Sciences, Beijing, China; 8 The Second Affiliated Hospital of Shenzhen University, Shenzhen, China; 9 Department of Epidemiology and Biostatistics, School of Public Health, Peking University, Beijing, China

## Abstract

**Importance:**

Type 2 diabetes (T2D) is associated with the progression of cognitive dysfunction. Physical activity benefits cognition, but no evidence from randomized clinical trials has shown whether tai chi chuan has better long-term benefits than fitness walking in cognitive function for patients with T2D and mild cognitive impairment (MCI).

**Objective:**

To compare the effectiveness of tai chi chuan, a mind-body exercise, for improving cognitive function in older adults with T2D and MCI, with fitness walking.

**Design, Setting, and Participants:**

This randomized clinical trial was conducted between June 1, 2020, and February 28, 2022, at 4 sites in China. Participants included 328 adults (aged ≥60 years) with a clinical diagnosis of T2D and MCI.

**Interventions:**

Participants were randomized in a 1:1:1 ratio to a tai chi chuan group, a fitness walking group, or a control group. The tai chi chuan group received 24-form simplified tai chi chuan. The fitness walking group received fitness walking training. Both exercise groups took the training for 60 min/session, 3 times/wk, for 24 weeks in a supervised setting. All 3 groups were provided with a 30-minute diabetes self-management education session, once every 4 weeks for 24 weeks. The participants were followed up for 36 weeks.

**Main Outcomes and Measures:**

The primary outcome was the global cognitive function measured at 36 weeks by the Montreal Cognitive Assessment (MoCA). Secondary outcomes included MoCA at 24 weeks and other cognitive subdomain measures and blood metabolic indices at 24 and 36 weeks.

**Results:**

A total of 328 participants (mean [SD] age, 67.55 [5.02] years; mean [SD] T2D duration, 10.48 [6.81] years; 167 [50.9%] women) were randomized to the tai chi chuan group (n = 107), fitness walking group (n = 110), or control group (n = 111) and included in the intention-to-treat analysis. At 36 weeks, the tai chi chuan group showed improved MoCA scores compared with the fitness walking group (mean [SD], 24.67 [2.72] vs 23.84 [3.17]; between-group mean difference, 0.84 [95% CI, 0.02-1.66]; *P* = .046) in the intention-to-treat analysis. The per-protocol analysis data set and subgroup analysis at 36 weeks showed similar results. Based on the generalized linear models, the treatment effects were similar in each group after adjusting for self-reported dietary calories and physical activity. There were 37 nonserious adverse events (tai chi chuan group, 8; fitness walking group, 13; control group, 16) unrelated to the study with no statistically significant difference among the 3 groups (*P* = .26).

**Conclusions and Relevance:**

In this randomized clinical trial including older adults with T2D and MCI, tai chi chuan was more effective than fitness walking in improving global cognitive function. The findings support a long-term benefit, suggesting the potential clinical use of tai chi chuan as an exercise intervention to improve cognitive function for older adults with T2D and MCI.

**Trial Registration:**

ClinicalTrials.gov Identifier: NCT04416841

## Key Points

**Question:**

Is tai chi chuan as a mind-body exercise intervention more effective than fitness walking for improving cognitive function for older adults with type 2 diabetes (T2D) and mild cognitive impairment (MCI)?

**Findings:**

In this randomized clinical trial including 328 adults 60 years or older with T2D and MCI, tai chi chuan showed significantly more benefit on global cognitive function at 36 weeks compared with fitness walking. There was no significant difference between the 2 exercise groups at 24 weeks.

**Meaning:**

These findings suggest that mindfulness tai chi chuan may be an effective cognitive treatment option for older adults with T2D and MCI.

## Teaser

This randomized clinical trial explores the effectiveness of tai chi chuan in improving cognitive function of older adults with type 2 diabetes and mild cognitive impairment (MCI) compared with a fitness walking group and a control group.

## Introduction

Type 2 diabetes (T2D) and cognitive dysfunction are highly prevalent and frequently coexist in older adults.^1^ Cognitive dysfunction is now accepted as an important and common comorbidity or even complication of diabetes.^2^ Cognitive decline might be associated with reduced self-care, increased use of care services, and greater dependency in diabetes.^1^ It can roughly be divided into 3 stages: diabetes-associated cognitive decrements, mild cognitive impairment (MCI), and dementia.^2^ Mild cognitive impairment is the transition stage between normal cognitive aging and dementia,^3^ which is also a critical window for intervention. The prevalence of MCI among individuals with T2D has been reported to be as much as 45%.^4^ Moreover, the rate of progression from MCI to dementia is 1.5 to 3.0 times higher in patients with T2D than in those without T2D.^5^ Although practice guidelines from both the American Diabetes Association and the UK multidisciplinary National Expert Working Group recommend paying attention to cognitive dysfunction in T2D, the evidence of clinical trials for nonpharmacological interventions is lacking.^1^

Exercise is an essential component of the management programs advised for patients with T2D.^6^ Evidence has shown that exercise has both acute and chronic effects on diabetes management.^7^ The American College of Sports Medicine recommended that regular physical activity potentially has psychological and cognitive function benefits for people with T2D.^8^ Combined exercise training can improve specific domains of cognitive functions in middle-aged and older adults with T2D.^9^ However, identifying optimal choices from these evidence-based cognitive interventions is challenging because there are few comparative effectiveness studies available, especially for older adults with T2D and MCI. Tai chi chuan is an increasingly popular multimodal mind-body exercise that incorporates physical, cognitive, social, and meditative components in the same activity^10^ and is therefore thought to promote brain health. A meta-analysis^11^ found that mind-body exercise, including tai chi chuan, was effective in controlling blood glucose levels in patients with T2D. Another study^12^ showed that tai chi chuan effectively improved cognitive function in older adults with MCI. Mind-body exercise, with an emphasis on mindfulness during physical effort, may be superior to conventional physical activity for cognitive management in older adults with T2D and MCI. However, no large-scale randomized clinical trials have been reported on whether tai chi chuan is more beneficial than fitness walking for patients with T2D and MCI.

Therefore, we designed a randomized clinical trial to explore the effectiveness of tai chi chuan in improving cognitive function of older adults with T2D and MCI compared with a fitness walking group and a control group. We hypothesized that tai chi chuan would play a better role in improving cognitive function of older adults with T2D and MCI.

## Methods

### Study Design

This multicenter randomized clinical trial had 3 parallel groups and was conducted at 4 sites in China (Fuzhou, Harbin, Shenzhen, and Beijing). The trial protocol and statistical analysis plan are available in Supplement 1. The local ethics committees for medical research at each study site approved the study. All participants provided written informed consent. The study followed the Consolidated Standards of Reporting Trial (CONSORT) reporting guideline.

### Participants

The study included community-dwelling adults from 4 sites. Enrollment began June 1, 2020, and ended February 28, 2022. Inclusion criteria were (1) a clinical diagnosis of T2D, (2) the presence of MCI without dementia, (3) 60 years or older, (4) no engagement in regular exercise in the last 3 months, and (5) informed consent and voluntary participation. Exclusion criteria included (1) cognitive impairment caused by other reasons, (2) presence of medical conditions that made exercise unsafe or the patient unable to exercise, and (3) participation in other experiments that influence this study.

### Randomization and Masking

**Figure 1.**
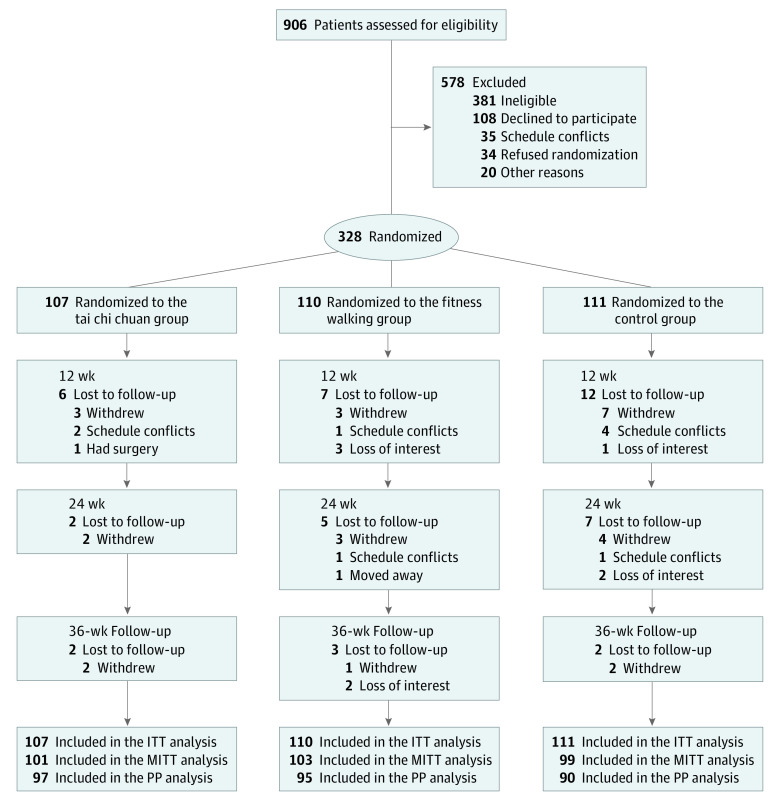
Study Flowchart ITT indicates intention to treat; MITT, modified ITT; and PP, per-protocol.

Research Electronic Data Capture (REDCap) data set system was used to randomly assign participants to the tai chi chuan, fitness walking, or control groups in a 1:1:1 ratio (Figure 1). Randomization was stratified by study sites with a block size of 6. Although blinding is not possible for participants in exercise-intervention research, the outcome assessors and data analysts were masked to group assignments. The study was unblinded after the statistical analyses were completed.

### Interventions

All groups participated in education seminars related to T2D management, including proper diet, blood glucose level monitoring, and prevention of complications, given by clinical endocrinologists for 0.5 hours each time, once every 4 weeks for 24 weeks. Participants who were assigned to the control group did not receive exercise intervention and maintained their previous lifestyle. Participants in the fitness walking group participated in a supervised 24-week fitness walking program. Participants in the tai chi chuan group received supervised 24-week, 24-form tai chi chuan training. Tai chi chuan is a form of mind-body exercise aiming at integrating musculoskeletal, sensory, and cognitive systems. It focuses on controlled, self-initialed exercise with synchronized breathing, and the movement patterns include center of gravity displacement, weight bearing and shifting, trunk and pelvic rotation, and eye-hand coordinated movements. Both intervention groups participated in 1-hour training sessions 3 times per week. The participants were also encouraged to continue exercise after completing their 24 weeks of supervised training, until the 36-week follow-up evaluation. The fitness walking and tai chi chuan training were instructed by certified instructors with at least 5 years of experience directing their respective interventions.

### Measurements and Outcomes

All participants were assessed at baseline and at 24 and 36 weeks. Assessments were conducted in accordance with a uniform implementation plan and standard operating procedures.

### Primary Outcomes

The primary outcome was global cognition assessed at 36 weeks. The Montreal Cognitive Assessment (MoCA)^13^ was used to assess global cognition. Scores on the MoCA ranged from 0 to 30, with higher scores indicating better cognitive function.

### Secondary Outcomes

Secondary outcomes included MoCA assessment at 24 weeks and other cognitive subdomain tests and blood metabolic indices measured at 24 and 36 weeks. Cognitive subdomain tests included the Wechsler Memory Quotient (MQ), Digit Symbol Substitution Test (DSST), Trail-Making Test, part B (TMT-B), Boston Naming Test (BNT), and Rey-Osterrieth Complex Figure Test (ROCF). Participants were asked to fast overnight and had their venous blood drawn on an empty stomach the next morning. Serum levels of fasting glucose, insulin, glycated hemoglobin (HbA_1c_), advanced glycation end products (AGE), and soluble receptor of AGE (sRAGE) were analyzed. The homeostasis model assessment of insulin resistance (HOMA-IR) was calculated as the product of fasting insulin level (in μIU/mL) and fasting glucose level (in mg/dL) divided by 22.5. Circulating levels of AGE and sRAGE were measured by enzyme-linked immunosorbent assay kits according to the manufacturer’s instructions (AGE: HUFI00449 [AssayGenie]; sRAGE: EK0827 [BosterBio]). AGE levels (in μg/mL) were divided by sRAGE levels (in pg/mL) to calculate the ratio (in μg/pg).

### Statistical Analysis

Based on a previous study^14^ and our pilot study, we used PASS software, version 15.0 (NCSS Statistical Software), to calculate that a sample size of approximately 109 participants would be needed in each group (considering a 20% dropout rate) to achieve an 80% statistical power and a 2-sided level of statistical significance at 5% for the comparisons of the 2 intervention groups (tai chi chuan and fitness walking) vs the control group at the primary end point. Comparisons between tai chi chuan and fitness walking groups were not performed due to a lack of trial data.

All the participants were included in the intention-to-treat (ITT) analysis. Modified ITT analysis included all randomized participants who completed at least 12 weeks of the intervention. Per-protocol analysis included participants who adhered to the treatment protocol. Safety set included the data collected for participants who received at least 1 intervention after randomization and for whom safety indicators were documented. Primary analyses were conducted using multiple imputation methods for missing observations at baseline, post intervention, and at follow-up, assuming missing data are missing at random. Prespecified sensitivity analyses were conducted for all participants using complete-case data at each time point. We also compared treatment effects in each group after adjusting for participant self-reported dietary calories and physical activity using a generalized linear model.

We report continuous variables as mean (SD) or median (IQR). We used 1-way analysis of variance or the Kruskal-Wallis test for between-group comparisons as appropriate. Categorical variables were described using numbers and percentages and analyzed using the χ^2^ test or the Fisher exact test. The primary analysis was to determine whether tai chi chuan training improved the MoCA score compared with fitness walking training and control at 36 weeks. These continuous outcomes were assessed by the 1-way analysis of variance model at 24 and 36 weeks. Given the large number of secondary outcomes, the secondary outcomes should be interpreted as exploratory. All statistical analyses were performed using SPSS, version 24.0 (IBM Corporation).

## Results

### Participant Characteristics

A total of 328 participants (mean [SD] age, 67.55 [5.02] years; T2D duration, 10.48 [6.81] years; 161 [49.1%] men and 167 [50.9%] women; all Chinese) were randomized to the tai chi chuan group (n = 107), fitness walking group (n = 110), and control group (n = 111) and included in the ITT analysis. Participant recruitment started on June 1, 2020, and the last participant completed the follow-up evaluation on February 28, 2022. From the 906 participants screened for eligibility, 328 were enrolled and randomized in the trial. Among these participants, 303 (92.4%) completed evaluations at 12 weeks, 289 (88.1%) completed evaluations at 24 weeks, and 282 (86.0%) completed evaluations at 36 weeks (Figure 1). All the participants were included in the ITT analysis, those who attended the 12-week evaluation were included in the modified ITT analysis, and those who attended evaluations at 36 weeks were included in the per-protocol analysis. The baseline demographic and clinical characteristics of participants are summarized in Table 1. Median number of diabetes medications, patient self-reported dietary calorie intake, and physical activity of the 3 groups by ITT are shown in eTable 1 in Supplement 2.

**Table 1.** Baseline Characteristics of Participants

Characteristic	Study group^a^		
	Tai chi chuan (n = 107)	Fitness walking (n = 110)	Control (n = 111)
Age, mean (SD), y	67.56 (4.99)	67.46 (4.73)	67.62 (5.35)
Sex			
Women	58 (54.2)	49 (44.5)	60 (54.1)
Men	49 (45.8)	61 (55.5)	51 (45.9)
BMI, mean (SD)	24.32 (3.03)	23.86 (2.90)	23.98 (3.40)
Disease duration, mean (SD), y	9.82 (5.58)	10.70 (7.49)	10.90 (7.20)
Educational duration, mean (SD), y	10.36 (3.27)	9.92 (3.59)	10.13 (3.47)
Comorbid illness, No.^b^			
≤1	85 (79.4)	86 (78.2)	84 (75.7)
>1	22 (20.6)	24 (21.8)	27 (24.3)
Smoking			
Never	79 (73.8)	71 (64.5)	87 (78.4)
Former	21 (19.6)	25 (22.7)	18 (16.2)
Current	7 (6.5)	14 (12.7)	6 (5.4)
Alcohol consumption			
Never	75 (70.1)	79 (71.8)	83 (74.8)
Former	18 (16.8)	8 (7.3)	13 (11.7)
Current	14 (13.1)	23 (20.9)	15 (13.5)
Blood pressure, mean (SD), mm Hg			
Systolic	130.59 (13.82)	130.52 (13.22)	131.46 (12.84)
Diastolic	79.27 (10.31)	80.17 (9.99)	78.71 (9.13)
Fasting glucose level, mg/dL	135.7 (42.3)	135.0 (40.0)	142.5 (42.0)
HbA_1c_ level, %	7.04 (1.20)	6.84 (1.41)	7.14 (1.48)
Log HOMA-IR, mean (SD)^c^	0.51 (0.25)	0.49 (0.29)	0.55 (0.26)
Diabetes medications			
Metformin	61 (57.0)	55 (50.0)	61 (55.0)
Sulfonylurea	20 (18.7)	26 (23.6)	22 (19.8)
Glinides	12 (11.2)	5 (4.5)	5 (4.5)
α-Glucosidase inhibitor	33 (30.8)	24 (21.8)	34 (30.6)
DPP-4 inhibitor	15 (14.0)	9 (8.2)	9 (8.1)
Insulin	18 (16.8)	25 (22.7)	20 (18.0)

Abbreviations: BMI, body mass index (calculated as weight in kilograms divided by height in meters squared); DPP-4, dipeptidyl peptidase 4; HbA_1c_, glycated hemoglobin; HOMA-IR, homeostasis model assessment of insulin resistance.

SI conversion factor: To convert fasting glucose to millimoles per liter, multiply by 0.0555. To convert HbA_1c_ to proportion, multiply by 0.01.

^a^ Unless otherwise indicated, data are expressed as No. (%) of participants. Percentages have been rounded and may not total 100.

^b^ Comorbidities were reported at screening. Selected comorbidities were presented based on a history of hypertension, coronary artery disease, chronic obstructive pulmonary disease, or dyslipidemia.

^c^ Calculated as [fasting plasma glucose level (in mg/dL)× fasting plasma insulin level (in μIU/mL) / 22.5]. Log-transformed data presented because HOMA-IR data did not meet the assumption of normality.

The overall intervention attendance rate across the 24 weeks was 95 (88.8%) in the tai chi chuan group and 99 (90.0%) in the fitness walking group (ie, attended ≥54 sessions, or >75.0% of the planned total intervention class sessions). The mean (SD) number of completed sessions was 63 (5) for both interventions groups, 64 (5) in the tai chi chuan group, and 63 (5) in the fitness walking group, with no significant difference between groups (*P* = .08).

### Primary Outcomes

In the ITT analysis, at 36 weeks, the increase of mean MoCA scores in the tai chi chuan group was significantly more noticeable than in the fitness walking group (24.67 [2.72] vs 23.84 [3.17]; between-group mean difference, 0.84 [95% CI, 0.02-1.66]; *P* = .046); In the per-protocol analysis, the tai chi chuan group was also significantly more effective in improving mean MoCA scores compared with the fitness walking group (24.87 [2.64] vs 23.93 [3.36], respectively; between-group mean difference, 0.94 [95% CI, 0.04-1.84]; *P* < .001) (Table 2). The modified ITT analysis demonstrated similar results as the ITT and per-protocol analyses (eTable 2 in Supplement 2). Based on generalized linear models, we found similar results after adjusting for self-reported dietary calorie intake and physical activity (eTable 3 in Supplement 2).

**Table 2.** Mean Difference in MoCA Score in 3 Groups by ITT and Per-Protocol Analyses

Measurement time	Study group			*P* value	Between-group change, mean (95% CI)		
	Tai chi chuan^a^	Fitness walking^b^	Control^c^		Tai chi chuan vs fitness walking	Tai chi chuan vs control	Fitness walking vs control
**ITT Analysis MoCA score, mean (SD)^d^**							
Baseline	21.38 (2.77)	21.52 (2.57)	21.34 (2.85)	.88	−0.14 (−0.86 to 0.59)	0.04 (−0.69 to 0.77)	0.18 (−0.55 to 0.90)
24 wk	23.99 (3.10)	23.55 (3.34)	22.54 (3.29)	.004	0.45 (−0.42 to 1.31)	1.45 (0.59 to 2.32)^e^	1.01 (0.15 to 1.86)^e^
36 wk	24.67 (2.72)	23.84 (3.17)	22.77 (3.29)	<.001	0.84 (0.02 to 1.66)^e^	1.90 (1.08 to 2.72)^e^	1.06 (0.25 to 1.87)^e^
**Per-protocol analysis MoCA score, mean (SD)^d^**							
Baseline	21.38 (2.79)	21.54 (2.44)	21.49 (2.78)	.92	−0.16 (−0.91 to 0.60)	−0.11 (−0.88 to 0.66)	0.05 (−0.73 to 0.82)
24 wk	24.13 (3.13)	23.53 (3.53)	22.63 (3.52)	.01	0.61 (−0.36 to 1.57)	1.50 (0.52 to 2.48)^e^	0.89 (−0.09 to 1.88)
36 wk	24.87 (2.64)	23.93 (3.36)	22.66 (3.47)	<.001	0.94 (0.04 to 1.84)^e^	2.21 (1.30 to 3.12)^e^	1.27 (0.35 to 2.19)^e^

Abbreviations: ITT, intention to treat; MoCA, Montreal Cognitive Assessment.

^a^ Includes 107 participants in the ITT analysis and 97 in the per-protocol analysis.

^b^ Includes 110 participants in the ITT analysis and 95 in the per-protocol analysis.

^c^ Includes 111 participants in the ITT analysis and 90 in the per-protocol analysis.

^d^ Scores range from 0 to 30, with higher scores indicating better cognitive function.

^e^ *P* < .05.

### Secondary Outcomes

#### Tai Chi Chuan vs Fitness Walking Groups

At 24 weeks, there was no statistical difference in MoCA score improvement between the tai chi chuan and fitness walking groups (Table 2). The tai chi chuan group was more effective in improving the mean DSST score compared with the fitness walking group (34.14 [10.79] vs 30.82 [9.67], respectively; between-group mean difference, 3.33 [95% CI, 0.63-6.03]), but there were no significant differences in other secondary outcomes (MQ, TMT-B, BNT, and ROCF scores, fasting glucose and HbA_1c_ levels, HOMA-IR, and AGE:sRAGE ratio) between the 2 groups (Table 3).

**Table 3.** Mean Difference of Secondary Outcomes in 3 Groups by ITT

ITT measurement	Study group			*P* value	Between-group change, mean (95% CI)		
	Tai chi chuan (n = 107)	Fitness walking (n = 110)	Control (n = 111)		Tai chi chuan vs fitness walking	Tai chi chuan vs control	Fitness walking vs control
**MQ, mean (SD)**^a^							
Baseline	90.04 (13.69)	89.01 (14.04)	87.41 (16.30)	.42	1.03 (−2.91 to 4.97)	2.62 (−1.31 to 6.55)	1.59 (−2.31 to 5.50)
24 wk	98.85 (10.41)	95.89 (13.28)	92.79 (14.13)	<.001	2.96 (−0.44 to 6.36)	6.06 (2.67 to 9.45)^b^	3.10 (−0.27 to 6.47)
36 wk	99.39 (12.7)	95.14 (14.37)	92.98 (14.43)	<.001	4.26 (0.55 to 7.96)^b^	6.41 (2.71 to 10.11)^b^	2.15 (−1.52 to 5.83)
**Digit Symbol Substitution Test score, mean (SD)^c^**							
Baseline	29.53 (9.91)	28.53 (9.21)	28.50 (9.02)	.65	1.01 (−1.50 to 3.51)	1.03 (−1.47 to 3.53)	0.03 (−2.46 to 2.51)
24 wk	34.14 (10.79)	30.82 (9.67)	29.81 (9.85)	.005	3.33 (0.63 to 6.03)^b^	4.33 (1.64 to 7.03)^b^	1.01 (−1.67 to 3.68)
36 wk	33.82 (10.48)	32.05 (9.89)	30.70 (9.60)	.07	1.77 (−0.90 to 4.44)	3.12 (0.46 to 5.78)	1.35 (−1.30 to 3.99)
**TMT-B finding, mean (SD), s^d^**							
Baseline	224.41 (95.16)	235.50 (99.40)	227.12 (82.36)	.65	−11.09 (−35.81 to 13.63)	−2.71 (−27.38 to 21.95)	8.37 (−16.12 to 32.87)
24 wk	202.25 (72.19)	206.78 (75.11)	223.86 (85.00)	.10	−4.54 (−25.29 to 16.22)	−21.62 (−42.33 to −0.91)	−17.08 (−37.65 to 3.48)
36 wk	187.76 (74.46)	206.62 (77.65)	215.53 (74.47)	.02	−18.86 (−39.04 to 1.32)	−27.78 (−47.91 to −7.64)^b^	−8.92 (−28.91 to 11.08)
**Boston Naming Test score, mean (SD)^e^**							
Baseline	22.46 (3.21)	22.58 (3.58)	21.82 (4.30)	.27	−0.12 (−1.23 to 0.99)	0.64 (−0.60 to 1.87)	0.76 (−0.52 to 2.04)
24 wk	24.73 (2.73)	24.86 (2.97)	24.04 (3.54)	.11	−0.13 (−1.06 to 0.8)	0.69 (−0.34 to 1.72)	0.82 (−0.24 to 1.87)
36 wk	25.37 (2.42)	25.30 (3.34)	24.73 (3.63)	.26	0.08 (−0.88 to 1.03)	0.64 (−0.36 to 1.65)	0.57 (−0.56 to 1.70)
**Rey-Osterrieth Complex Figure Test score, mean (SD)^f^**							
Baseline	32.21 (5.9)	31.95 (6.09)	31.84 (5.95)	.90	0.26 (−1.33 to 1.86)	0.37 (−1.23 to 1.96)	0.10 (−1.48 to 1.69)
24 wk	33.01 (5.53)	32.3 (5.62)	32.68 (3.89)	.58	0.72 (−0.64 to 2.07)	0.33 (−1.02 to 1.68)	−0.39 (−1.73 to 0.95)
36 wk	32.76 (6.25)	31.74 (6.63)	32.36 (5.00)	.45	1.02 (−0.58 to 2.62)	0.40 (−1.19 to 2.00)	−0.62 (−2.21 to 0.97)
**Rey-Osterrieth Complex Figure Test delayed recall score, mean (SD)^f^**							
Baseline	13.81 (7.71)	13.75 (6.87)	13.59 (7.13)	.97	0.05 (−1.88 to 1.99)	0.22 (−1.71 to 2.15)	0.17 (−1.75 to 2.09)
24 wk	17.9 (8.21)	18.26 (8.01)	18.16 (8.44)	.95	−0.37 (−2.56 to 1.83)	−0.26 (−2.45 to 1.93)	0.11 (−2.07 to 2.28)
36 wk	18.97 (8.35)	18.91 (8.15)	18.87 (8.53)	.99	0.06 (−2.17 to 2.29)	0.1 (−2.12 to 2.32)	0.04 (−2.17 to 2.25)
**Fasting glucose level, mean (SD), mg/dL**							
Baseline	135.7 (42,3)	135.0 (40.0)	142.5 (42.2)	.32	0.7 (−10.5 to 11.7)	−6.9 (−18.0 to 4.1)	−7.6 (−18.6 to 3.4)
24 wk	132.1 (32.1)	138.2 (36.6)	139.5 (38.4)	.27	−6.1 (−15.7 to 3.4)	−7.4 (−16.9 to 2.2)	−1.3 (−10.6 to 8.3)
36 wk	129.4 (25.9)	139.6 (36.2)	140.0 (29.7)	.02	−10.3 (−18.6 to −2.0)^b^	−10.6 (−18.9 to −2.3)^b^	−0.4 (−8.5 to 7.9)
**HbA_1c_ level, mean (SD), %**							
Baseline	7.04 (1.2)	6.84 (1.41)	7.14 (1.48)	.25	0.20 (−0.17 to 0.57)	−0.10 (−0.47 to 0.26)	−0.30 (−0.66 to 0.06)
24 wk	6.88 (1.06)	6.91 (1.20)	7.13 (1.25)	.24	−0.03 (−0.34 to 0.29)	−0.25 (−0.56 to 0.07)	−0.22 (−0.53 to 0.09)
36 wk	7.08 (1.10)	7.03 (1.09)	7.25 (1.17)	.31	0.05 (−0.25 to 0.35)	−0.17 (−0.47 to 0.13)	−0.22 (−0.52 to 0.08)
**Log HOMA-IR, mean (SD)^g^**							
Baseline	0.51 (0.25)	0.49 (0.29)	0.55 (0.26)	.20	0.02 (−0.05 to 0.1)	−0.04 (−0.11 to 0.03)	−0.06 (−0.13 to 0.01)
24 wk	0.42 (0.24)	0.45 (0.28)	0.46 (0.26)	.44	−0.03 (−0.10 to 0.04)	−0.04 (−0.11 to 0.02)	−0.02 (−0.09 to 0.05)
36 wk	0.40 (0.25)	0.43 (0.22)	0.46 (0.26)	.15	−0.03 (−0.10 to 0.03)	−0.06 (−0.13 to 0.00)	−0.03 (−0.09 to 0.04)
**AGE:sRAGE ratio, median (IQR), μg/pg**							
Baseline	0.16 (0.14)	0.16 (0.15)	0.14 (0.15)	.55	0.00 (−0.04 to 0.04)	0.02 (−0.02 to 0.06)	0.02 (−0.02 to 0.06)
24 wk	0.06 (0.05)	0.06 (0.05)	0.06 (0.04)	.91	0.00 (−0.01 to 0.02)	0.00 (−0.01 to 0.02)	0.00 (−0.01 to 0.02)
36 wk	0.05 (0.03)	0.07 (0.05)	0.07 (0.04)	<.001	−0.03 (−0.04 to −0.01)^b^	−0.02 (−0.03 to −0.01)^b^	0.01 (−0.01 to 0.02)

Abbreviations: AGE:sRAGE, advanced glycation end products/soluble receptor for advanced glycation end products; HbA_1c_, glycated hemoglobin; HOMA-IR, homeostasis model assessment of insulin resistance; ITT, intention to treat; MQ, Wechsler Memory Quotient; TMT-B, Trail Making Test part B.

SI conversion factor: To convert fasting glucose to millimoles per liter, multiply by 0.0555. To convert HbA_1c_ to proportion, multiply by 0.01.

^a^ Scores range from 51 to 151, with higher scores indicating better cognitive function.

^b^ *P* < .05.

^c^ Scores range from 0 to 133, with higher scores indicating better cognitive function.

^d^ Scored as completion time.

^e^ Scores range from 0 to 30, with higher scores indicating better cognitive function.

^f^ Scores range from 0 to 36, with higher scores indicating better cognitive function.

^g^ Calculated as (fasting plasma glucose level [in mg/dL] × fasting plasma insulin level [in μIU/mL] / 22.5). Log-transformed data presented because HOMA-IR data did not meet the assumption of normality.

At 36 weeks, compared with the fitness walking group, the tai chi chuan group was significantly more effective in improving mean MQ scores (99.39 [12.70] vs 95.14 [14.37], respectively; between-group mean difference, 4.26 [95% CI, 0.55-7.96]), mean AGE:sRAGE ratio (0.05 [0.03] vs 0.07 [0.05], respectively; between-group mean difference, −0.02 [95% CI, −0.03 to −0.01]), and mean fasting glucose level (129.4 [25.9] vs 139.5 [36.2] mg/dL, respectively; between-group mean difference, −10.3 [95% CI, −18.6 to −2.3] mg/dL [to convert to millimoles per liter, multiply by 0.0555]) (Table 3). There were no significant differences in other secondary outcomes (DSST, TMT-B, BNT, and ROCF scores, HbA_1c_ level, and HOMA-IR) between the tai chi chuan and fitness walking groups (Table 3).

#### Tai Chi Chuan vs Control Groups

At 24 weeks, the tai chi chuan group was significantly more effective in improving mean MoCA scores compared with the control group (23.99 [3.10] vs 22.54 [3.29]; between-group mean difference, 1.45 [95% CI, 0.59-2.32]) in ITT analysis (Table 2). The tai chi chuan group was significantly more effective in improving mean MQ scores compared with the control group (98.85 [10.41] vs 92.79 [14.13], respectively; between-group mean difference, 6.06 [95% CI, 2.67-9.45]) (Table 3). Compared with the control group, the tai chi chuan group was significantly more effective in improving mean DSST scores (34.14 [10.79] vs 29.81 [9.85], respectively; between-group mean difference, 4.33 [95% CI, 1.64-7.03]) and TMT-B scores (202.25 [72.19] vs 223.86 [85.00], respectively; between-group mean difference, −21.62 [95% CI, −42.33 to −0.91]) (Table 3). There were no significant differences in other secondary outcomes (BNT and ROCF scores, fasting glucose and HbA_1c_ levels, HOMA-IR, and AGE:sRAGE ratio) between the tai chi chuan and control groups (Table 3).

At 36 weeks, compared with the control group, the tai chi chuan group was significantly more effective in improving mean MQ scores (99.39 [12.70] vs 92.98 [14.43], respectively; between-group mean difference, 6.41 [95% CI, 2.71-10.11]), DSST scores (33.82 [10.48] vs 30.70 [9.60], respectively; between-group mean difference, 3.12 [95% CI, 0.46-5.78]), TMT-B scores (187.76 [74.46] vs 215.53 [74.47], respectively; between-group mean difference, −27.78 [95% CI, −47.91 to −7.64]), fasting glucose level (129.4 [25.9] vs 140.0 [29.7] mg/dL, respectively; between-group mean difference, −10.6 [95% CI, −18.9 to −2.3] mg/dL), and the AGE:sRAGE ratio (0.05 [0.03] vs 0.07 [0.04], respectively; between-group mean difference, −0.02 [95% CI, −0.03 to −0.01]) (Table 3). There were no significant differences in other secondary outcomes (BNT and ROCF scores, HbA_1c_ level, and HOMA-IR) between the tai chi chuan and control groups (Table 3).

#### Fitness Walking vs Control Groups

At 24 weeks, the fitness walking group was significantly more effective in improving mean MoCA scores compared with the control group (23.55 [3.34] vs 22.54 [3.29], between-group mean difference, 1.01 [95% CI, 0.15-1.86]) (Table 2). At 36 weeks, the fitness walking group was significantly more effective in improving mean MoCA scores compared with the control group (23.84 [3.17] vs 22.77 [3.29], respectively; between-group mean difference, 1.06 [95% CI, 0.25-1.87]) (Table 2). There were no significant differences in other outcomes (MQ, DSST, TMT-B, BNT, and ROCF scores, fasting glucose and HbA_1c_ levels, HOMA-IR, and AGE:sRAGE ratio) between the fitness walking and control groups at both 24 and 36 weeks (Table 3).

### Subgroup Analysis

**Figure 2.**
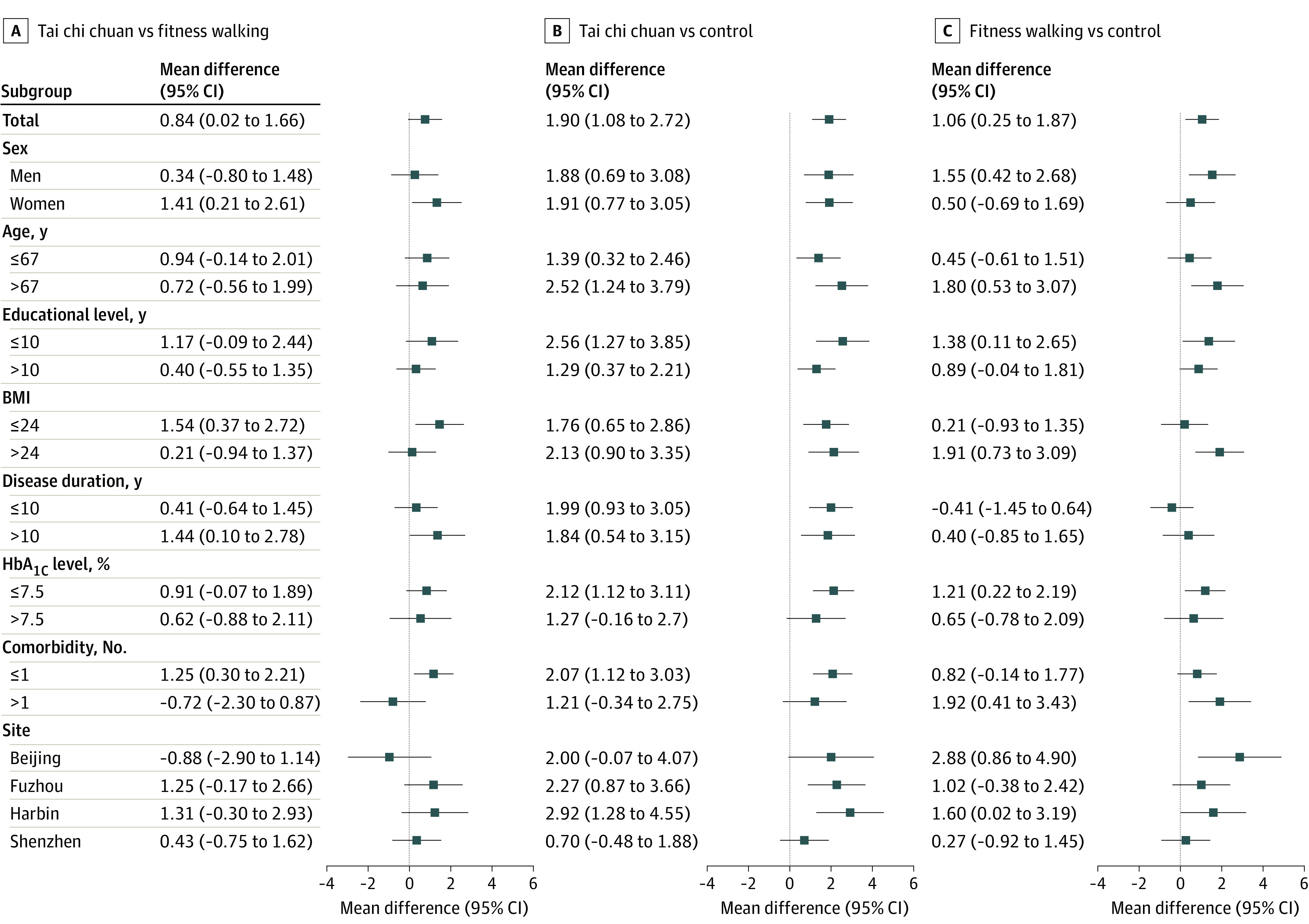
Subgroup Analysis of Montreal Cognitive Assessment (MoCA) at 36 Weeks by Intention-to-Treat (ITT) Analysis BMI indicates body mass index (calculated as weight in kilograms divided by height in meters squared); HbA_1c_, glycated hemoglobin (to convert to proportion, multiply by 0.01).

Subgroup analysis at 36 weeks showed that tai chi chuan group was significantly more effective in improving MoCA scores compared with fitness walking group in subgroups of women (mean difference, 1.41 [95% CI,0.21-2.61]), body mass index (BMI; calculated as weight in kilograms divided by height in meters squared) of 24.00 or less (mean difference, 1.54 [95% CI, 0.37-2.72]), T2D duration greater than 10 years (mean difference, 1.44 [95% CI, 0.10-2.78]), and 1 or fewer comorbidities (mean difference, 1.25 [95% CI, 0.30-2.21]) (Figure 2). Subgroup analysis at 24 weeks showed that tai chi chuan group was significantly more effective in improving mean MoCA scores compared with fitness walking group in the participants with BMI of 24.00 or less (mean difference, 1.56 [95% CI, 0.35 to 2.78]) (eFigure in Supplement 2).

### Adverse Events

Hypoglycemia events occurred in 1 participant (0.9%) in the tai chi chuan group and in 2 participants (1.8%) in the fitness walking group. Falls were reported in 5 participants (4.7%) in the tai chi chuan group, 10 (9.1%) in the fitness walking group, and 15 (13.5%) in the control group. One participant (0.9%) reported hospital admission in the control group and 1 participant (0.9%) required an emergency department visit in the tai chi chuan group. There was no statistically significant difference for total adverse events among 3 groups (*P* = .26). A diabetes specialist and a general practitioner used a standardized reporting system to evaluate these events for relevance to the intervention and severity. A complete listing of reported adverse events is provided in eTable 4 of Supplement 2.

## Discussion

To our knowledge, this randomized clinical trial is the first to show that tai chi chuan, a mind-body exercise, improved cognitive function for older adults with T2D and MCI compared with fitness walking. Among the participants, the MoCA and MQ scores in the tai chi chuan group were significantly higher than in the fitness walking and control groups at 36 weeks. Also, the tai chi chuan group had significantly better effects on AGE:sRAGE ratio and fasting blood glucose levels than the fitness walking group. Twenty-four weeks of tai chi chuan training significantly improved attention function in the tai chi chuan group compared with the fitness walking group.

Previously, the efficacy of tai chi chuan for patients with T2D and MCI was unclear. Even though physical activity was thought to be beneficial for cognitive function in patients with diabetes,^15^ randomized clinical trials^9,16,17^ showed that the effects of physical activity on global cognitive function and cognitive subdomains were inconsistent. In the present study, both tai chi chuan and fitness walking improved MoCA scores compared with the control group at 24 weeks. Although there was no significant difference in MoCA scores between the tai chi chuan and fitness walking groups at 24 weeks, the mean changes in the tai chi chuan group were greater than in the fitness walking group. Because tai chi chuan is a mind-body exercise that requires constant memorization and learning of movements, it took longer for patients to achieve better performance, which might be an important reason why the significant differences were found at 36 rather than 24 weeks.

A previous study^18^ showed that the MoCA scores of patients with MCI decreased by 0.52 points each year. Our study concluded that in the tai chi chuan group, MoCA scores improved by 1.90 points compared with the control group and by 0.84 points compared with the fitness walking group. Although an additional 0.84-point increase may seem minimal, the finding that tai chi chuan could improve MoCA scores by about 1 point in 9 months compared with fitness walking is a promising one. If the participants were able to continue practicing tai chi chuan exercise by themselves, they might see sustained improvements in cognitive function. These findings support the clinical use of tai chi chuan as an exercise intervention to improve cognitive function in older adults with T2D and MCI.

This randomized clinical trial found that tai chi chuan could improve global cognitive and memory functions, consistent with recently published systematic reviews and meta-analysis.^19,20^ There are several possible mechanisms for tai chi chuan to improve cognitive function. First, tai chi chuan is a moderate-intensity aerobic exercise with approximate 4.0 metabolic equivalents,^21^ overlapping with brisk walking, that showed positive effects on cognitive function.^22^ Importantly, studies have shown that tai chi chuan is beneficial to the structure and function of the brain regions, including the prefrontal cortex, temporal cortex, and hippocampus, all critical to the regulation of cognitive function and memory function.^23,24^ Besides, tai chi chuan training involves learning movements, memorization, concentration, and multitasking. This will highly integrate and coordinate mind, breathing, and movement and may stimulate synaptic building effectively.^20,22^ The meditation and relaxation training of tai chi chuan could reduce anxiety and depression, which may improve cortisol levels and other stress-related pathways of cognitive decline.^22^ In addition, tai chi chuan includes not only the component of aerobic exercise but also the components of muscle-strengthening activity and balance training. Tai chi chuan was effective in improving muscle strength and balance capacity, thereby reducing the incidence of falls among older adults.^25,26^ The muscle-strengthening components of tai chi chuan induced the release of neurochemicals such as brain-derived neurotrophic factor, insulinlike growth factor 1, and homocysteine, which had a positive effect on cognitive function.^27^ The muscle tension and contraction through tai chi chuan training might cause more metabolic stress and molecule release–associated cognitive changes.^28^ These elements of tai chi chuan may provide a greater effect in enhancing global cognitive and memory functions compared with walking.

We conducted a prespecified subgroup analysis of factors associated with T2D and cognitive impairment. The results showed that at 36 weeks, the tai chi chuan group performed better than the fitness walking group in both the global cognitive function and memory function for women and participants with BMI of 24.00 or less, T2D duration greater than 10 years, and 1 or fewer comorbidities. This finding is important because previous research has shown that women have a higher prevalence of dementia than men.^29^ The findings on duration of T2D, BMI,^30,31^ and comorbidity suggest that interventions may be more effective in the early or mild stages of the disease. Therefore, this also suggests that in the future, early tai chi chuan training would be more effective for people with T2D and MCI, especially women with low levels of education.

### Strengths and Limitations

This study has important strengths. Compared with the previous study,^9^ we recruited a larger sample size from multiple centers, which enhanced the generalizability. Our highly supervised exercise intervention had a high adherence rate and a low rate of loss to follow-up. Another strength is the selection of multiple validated cognitive tests to assess different cognitive domains. We performed ITT, modified ITT, and per-protocol analyses, and all the analyses of the primary outcome yielded similar effect estimates.

This study has several limitations. First, the study population was limited by the eligibility criteria used. Second, compared with the control group, participants who were assigned to exercise intervention groups might have anticipated the benefits of exercise and may have introduced bias into the results. Third, the follow-up period was relatively short, so we did not explore a longer-term treatment effect. Finally, the study was time-consuming and required extensive human resources; and future studies should consider how to improve participants’ adherence within the limited time and human resources so as to better promote clinical application.

## Conclusions

In this randomized clinical trial including older adults with T2D and MCI, tai chi chuan was found to be more effective than fitness walking at improving global cognitive function. The findings support a long-term benefit of tai chi chuan in strengthening cognitive function, supporting the clinical application of tai chi chuan as an exercise intervention to promote cognitive function for older adults with T2D and MCI.

## Notes

Supplement 1.
Trial Protocol and Statistical Analysis Plan

Supplement 2. **eTable 1.** Medians (IQR) of Diabetes Medication, Patient Self-reported Dietary Calories, and Physical Activity of 3 Groups by ITT **eTable 2.** Mean Difference (SD) of MoCA in 3 Groups by MITT **eTable 3.** Generalized Linear Model of MoCA and Physical Activity by ITT **eTable 4.** Safety Outcomes: Adverse Events **eFigure.** Subgroup Analysis of MoCA in 24 Weeks by ITT

Supplement 3.
Data Sharing Statement
