# Supplementary material for: Effects of Tai Chi Chuan on Cognitive Function in Adults 60 Years or Older With Type 2 Diabetes and Mild Cognitive Impairment in China: A Randomized Clinical Trial
Source: JAMA Netw Open. 2023 Apr 6;6(4):e237004. doi: 10.1001/jamanetworkopen.2023.7004 (PMC10080376; doi:10.1001/jamanetworkopen.2023.7004)
Supplement: Supplement 1. — Trial Protocol and Statistical Analysis Plan [file jamanetwopen-e237004-s001.pdf]

Effects of Tai chi Chuan on cognitive function in Adults 60 years old or older with type 2 diabetes and mild cognitive impairment: A Randomized Clinical trial

Study Protocol

Introduction

Type 2 diabetes mellitus (T2DM) is a chronic metabolic disorder characterized by sustained hyperglycemia and insulin resistance[1]. According to the International Diabetes Federation, the number of patients in China of having diabetes mellitus was about 1.409 million, which represented a sharp increase compared to the results of the same survey conducted in 2019 (1.289 million)[2], nearly 95% of diabetic cases are T2DM[3]. Cognitive impairment is a common comorbidity of diabetes, especially in elderly populations[4]. Mild cognitive impairment (MCI) is a prodromal stage of Alzheimer's disease[5]. Our previous Meta analysis found that about 45% of people with diabetes have MCI[6]. The rate of conversion from MCI to dementia is 1.5-3 times higher in diabetic patients than in non-diabetic patients[7]. Impaired cognitive function may cause impaired self-management in people with diabetes, worsening their condition.

It is critically important to identify effective treatments to enhance cognitive function of T2DM patients with MCI. Exercise is typically one of the first management strategies advised for patients with T2DM[8]. Tai Chi Chuan, a Chinese traditional mind-body exercise, incorporates physical, cognitive, social, and meditative components in the same activity[9]. Research shows that Tai chi Chuan is to be helpful in global cognitive function, memory and learning, visuospatial ability, and executive functions of MCI[10], and metabolic control of T2DM[11]. However, the evidence of the effect of Tai Chi Chuan on T2DM patients with MCI is limited. Therefore, we designed a multi-centre, randomised, parallel controlled clinical trial to explore the effect of Tai Chi Chuan treating T2DM patients with MCI in cognitive function.

Methods

Participants

Diagnostic criteria

The diagnosis of T2DM is based on the 2018 American Diabetes Association and World Health Organization criteria[12, 13]. MCI is diagnosed using the criteria of Petersen[14].

Inclusion criteria

We will include eligible participants: (1) clinician diagnosis of T2DM; (2) presence of mild cognitive impairment, not demented; (3) age ≥ 60 years old; (4) did not engage in regular exercise in the last three months (at least 3 times a week, at least 20 minutes of regular exercise each time); (5) informed consent and voluntary participation.

T2DM diagnosis criteria:

According to the American Diabetes Association diagnosis criteria for T2DM: FPG ≥126 mg/dL (7.0 mmol/L). Fasting is defined as no caloric intake for at least 8 h; or 2-h PG ≥200 mg/dL (11.1 mmol/L) during OGTT. The test should be performed as described by WHO, using a glucose load containing the equivalent of 75 g anhydrous glucose dissolved in water; OR A1C ≥6.5% (48 mmol/mol). The test should be performed in a laboratory using a method that is NGSP certified and standardized to the DCCT assay; OR In a patient with classic symptoms of hyperglycemia or hyperglycemic crisis, a random plasma glucose ≥200 mg/dL (11.1 mmol/L).

(DCCT, Diabetes Control and Complications Trial; FPG, fasting plasma glucose; OGTT, oral glucose tolerance test; WHO, World Health Organization; 2-h PG, 2-h plasma glucose.)

MCI diagnosis criteria：

According to the Peterson criteria for MCI: Cognitive impairment is confirmed by patients or informed persons or experienced clinicians; There is one or more cognitive aspects impairment (memory, language, visuospatial or executive function); Functional activities are basically normal; No dementia.

Exclusion criteria

Patients will be excluded who: (1) cognitive impairment caused by other reasons, taking drugs, poisoning, etc; (2) presence of medical conditions that unable or unsafe to exercise, such as depression symptoms, uncontrolled hypertension/ blood pressure/ blood glucose, nervous system diseases(stroke, Parkinson's disease, etc), musculoskeletal system diseases(arthritis, history of hip and/or knee joint replacement, etc), etc; (3) participating in other experiments that influence this study.

Sample size

Based on a previous study and a pilot study we did[15], A sample size of approximately 109 participants per group calculated using PASS (considering 20% dropout rate) was determined to provide 80% power to achieve statistical significance at the 5% 2-sided level for comparisons of

the two intervention groups (Tai Chi Chuan and fitness walking) versus control across primary end point. Comparisons between Tai Chi Chuan and fitness walking were not made due to lack of trial data.

Randomization and concealment

This study was a multicenter, randomized controlled clinical trial. Subjects were randomized 1:1:1 into Tai Chi Chuan group, fitness walking group, and control group using a central web-based randomization system for group randomization (group size of 6). Because blinding is not possible for participants in exercise-intervention researches, the study outcome assessors and statistical analysts will have no knowledge of the participants' study group. Blinding was discovered after the statistical analysis of the study was completed.

Ethics

The study adheres to the Helsinki Declaration. The clinical trial has been approved by the Regional Committee on Medical Research Ethics from four partner institution: (1) The Second People's Hospital Affiliated to Fujian University of Traditional Chinese Medicine, Fuzhou, Fujian, China (approval No. 2020KY-004-02), (2) The Second People's Hospital

Affiliated to Heilongjiang University of Traditional Chinese Medicine (approval No.2020-K24), (3) Xiyuan Hospital of China Academy of Chinese Medical Sciences, Beijing, China (approval No. 2020XLA033-2), and (4) Shenzhen Bao'an District People's Hospital, Shenzhen, China (approval No.BYL20200409). All participants provided written informed consent. Furthermore, a Data Security and Monitoring Committee has been established. The study was registered with https://clinicaltrials.gov/. (Register Number: NCT04416841).

Recruitment

Volunteers will be recruited from the communities of Fuzhou of Fujian Province, Harbin of Heilongjiang Province, Beijing Municipality, and Shenzhen of Guangdong Province. We will contact each patient, invite them to participate, and schedule an individual appointment after a combination of advertisements and enrollment through clinics. Patients who are interested in participating will be given an explanation of the study and will be able to choose whether or not to participate voluntarily. If they agree to participate in the study, a clinical research coordinator (CRC) will evaluate their suitability using inclusion and exclusion criteria. Eligible subjects will sign an informed consent form before being randomly assigned to one of three groups: Tai Chi Chuan, fitness walking, or control. The flowchart is shown in Figure 1.

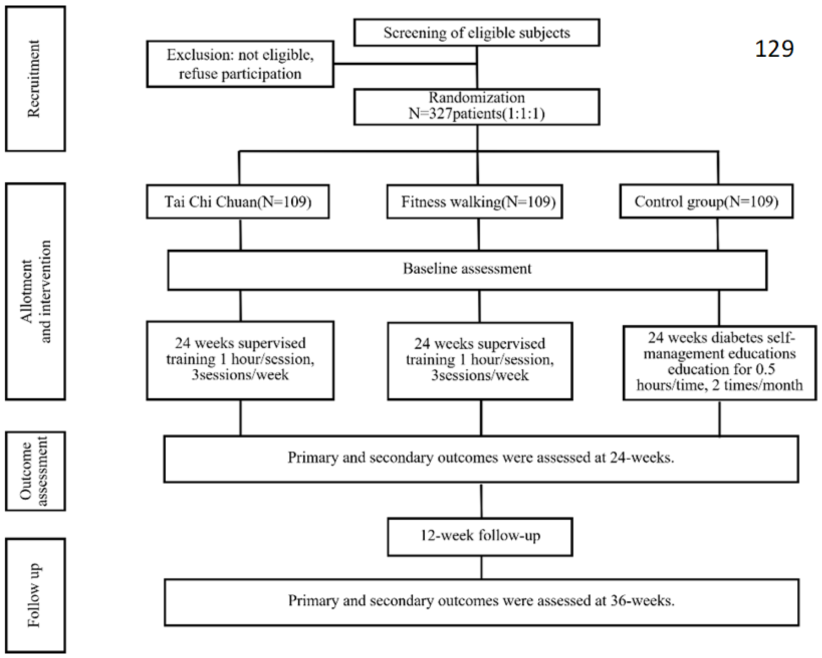

Figure 1. Flow chart for patient inclusion and follow up

Interventions

Clinical endocrinologist will give a lecture on the subject to all subjects, including diabetes knowledge, proper diet, blood glucose monitoring, and complications prevention, etc., for 0.5 hours per time, once per 4weeks for 24 weeks. In addition, participants assigned to the Tai Chi Chuan and fitness walking groups will engage in one hour of exercise three times a week for 24 weeks under the supervision of physical education instructors with more than five years of teaching experience. All sessions will include:

a. Warm-up (10 min)

b. Aerobic component (40 min):

Tai Chi Chuan group: would take 24-form simplified Tai Chi Chuan.

Fitness walking group: would take fitness walking training, the exercise intensity was 50% to 70% of the maximum heart rate.

c. Cool-down (10 min)

Participants who were assigned to control group did not received exercise intervention and maintained their previous lifestyle. The participants were also encouraged to continue exercise after completing their 24 weeks interventions, throughout 36 weeks of follow up.

Outcomes

Outcomes will be evaluated at baseline, 24 weeks, and 36 weeks. The primary end point was Montreal cognitive assessment scale (MOCA) at 36 weeks. Secondary outcomes include the primary outcome at 24 weeks, as well as other cognitive subdomain tests and blood metabolic indices at 24 and 36 weeks. Cognitive subdomain tests include: (1) Wechsler memory scale (WMS); (2) Digit Symbol Substitution Test (DSST); (3) Trial making test part B (TMT-B); (4) Boston naming test (BNT); (5) Rey-osterrieth complex graphics test (ROCF). Blood metabolic indices

included fasting glucose, glycated hemoglobin (HbA1c), homeostasis model assessment of insulin resistance (HOMA-IR), advanced glycation end products/soluble receptor of advanced glycation end products (AGE/sRAGE).

Measure

Demographics

At the first assessment, data on demographic characteristics will be collected: gender, age, years of education, duration of diabetes, diabetes medication, and history of disease, etc. were gathered by self-report. Height and weight were measured on site. Subjects were asked to wear light clothing and no shoes to record weight to the nearest 0.1 kg. Height was recorded to the nearest 0.5 cm. Body Mass Index (BMI) was calculated as weight (kilograms) divided by the square of height (meters).

cognitive performance measures

MOCA[16]*:* This scale is an assessment tool for global cognition. Scores range from 0 to 30, with higher scores indicating better cognitive functioning and scores below 26 indicating MCI.

WMS[17]*:* We used the WMS for evaluation of memory function.

Wechsler's Memory Quotient (MQ) has a score range of 51 to 150, with higher scores are considered to represent better memory.

DSST[18]*:* The Test test assesses attention and consists of a pairing of numbers and symbols. Symbols occur in the upper boxes and numbers in the lower boxes of a paired key. Participants then choose a target symbol that corresponds to a number, The number of correct numbers chosen within 90 seconds constitutes the score.

TMT-B[19]*:* We used the TMT-B for evaluation of executive function, The TMTB includes the numbers 1-25 in circles and squares and requires the participant to arrange the numbers from smallest to largest as quickly as possible while ensuring that the two shapes are connected alternately, and the evaluator records both the number of connection errors and the time, with shorter completion times and fewer connection errors indicating better performance.

BNT[20]*:* The BNT was used to assess language fluency. Subjects were asked to name the 30 objects/items printed on the card and the assessor recorded the number of correct names.

ROCF[21]*:* Copy and delayed recall will be administered as a measure of

graphic memory function. Patients were asked to copy the ROCF as accurately as possible on a piece of paper. after an interval of 20 minutes, patients were asked to recall and draw the graph optimally.

Blood metabolism index

Subjects were asked to fast overnight and venous blood was drawn on an empty stomach the next morning. Blood levels of fasting glucose, HbA1c, insulin, AGE, and sRAGE were analyzed. HOMA-IR index calculated from intravenous glucose and insulin levels: HOMA-IR = [fasting insulin (μIU/ml) × fasting glucose (mmol/L)]/22.5[22]. Aliquots of serum will be stored at -80'C and used for AGE and sRAGE assays. Serum concentrations of AGE and sRAGE were measured by ELISA kits according to the manufacturer's instructions (AGE:HUFI00449, Human AGE ELISA Kit, ReagentGenie, Ireland; RAGE: EK0827, Human RAGE ELISA Kit, Boster, China). All assays will be performed according to the manufacturer's instructions. AGE levels (μg/ml) were divided by sRAGE levels (pg/ml) to gain the ratio (μg/pg)[23].

Data management

All participant data will be identified by a study ID for enrolled participants All data from the completed CRFs will be imported into the

excel spreadsheet twice by two independent data entry officers in the proper format. This will be checked by another person to ensure its accuracy. Any errors made during data entry will be corrected by comparison with the original data. After completing the examination of the data, the data is imported into specific analysis software for analysis.

Statistical Analysis

Statistical analysis will be used for all data. All collected data (e.g. MOCA, WMS, DSST, TMT-B, BNT, ROCF, etc.) will be compared for all three groups. See the statistical analysis plan for details. Results with $P < 0.05$ will be considered statistically significant.

Safety

Throughout the course of the trial, each participant's safety will be watched over. All adverse events will be recorded, such as the number of times hypoglycemia occurs, falls, etc. and the relevance to treatment was evaluated by professional researchers.

Quality Control

Researchers receive uniform and strict training and examination. The trail

will be conducted in accordance with a uniform implementation plan and standard operating procedures. A designated project manager at each centre will be responsible for the quality of the research. Trial supervision provides quality control throughout, with regular visits to each centre to check the extent to which the trial has been conducted and whether the protocol has been strictly followed. At the end of the trial, all data will be checked by the quality controller, principal investigator and statistician.

Reference

1. Li, C.; Jin, R.; Liu, K.; Li, Y.; Zuo, Z.; Tong, H.; Zhang, J.; Zhang, J.; Guo, Y.; Lai, Y.; Sun, J.; Wang, J.; Xiong, K.; Chen, X., White Matter Atrophy in Type 2 Diabetes Mellitus Patients With Mild Cognitive Impairment. *Front Neurosci* **2020,** *14*, 602501.
2. Sun, H.; Saeedi, P.; Karuranga, S.; Pinkepank, M.; Ogurtsova, K.; Duncan, B. B.; Stein, C.; Basit, A.; Chan, J. C. N.; Mbanya, J. C.; Pavkov, M. E.; Ramachandaran, A.; Wild, S. H.; James, S.; Herman, W. H.; Zhang, P.; Bommer, C.; Kuo, S.; Boyko, E. J.; Magliano, D. J., IDF Diabetes Atlas: Global, regional and country-level diabetes prevalence estimates for 2021 and projections for 2045. *Diabetes Res Clin Pract* **2022,** *183*, 109119.
3. Saeedi, P.; Petersohn, I.; Salpea, P.; Malanda, B.; Karuranga, S.; Unwin, N.; Colagiuri, S.; Guariguata, L.; Motala, A. A.; Ogurtsova, K.; Shaw, J. E.; Bright, D.; Williams, R., Global and regional diabetes prevalence estimates for 2019 and projections for 2030 and 2045: Results from the International Diabetes Federation Diabetes Atlas, 9(th) edition. *Diabetes Res Clin Pract* **2019,** *157*, 107843.
4. Liu, J.; Yang, W.; Luo, H.; Ma, Y.; Zhao, H.; Dan, X., Brain-derived neurotrophic factor Val66Met polymorphism is associated with mild cognitive impairment in elderly patients with type 2 diabetes: a case-controlled study. *Aging Clin Exp Res* **2021,** *33* (6), 1659-1666.
5. Petersen, R. C.; Lopez, O.; Armstrong, M. J.; Getchius, T. S. D.; Ganguli, M.; Gloss, D.; Gronseth, G. S.; Marson, D.; Pringsheim, T.; Day, G. S.; Sager, M.; Stevens, J.; Rae-Grant, A., Practice guideline update summary: Mild cognitive impairment: Report of the Guideline Development, Dissemination, and Implementation Subcommittee of the American Academy of Neurology. *Neurology* **2018,** *90* (3), 126-135.
6. You, Y.; Liu, Z.; Chen, Y.; Xu, Y.; Qin, J.; Guo, S.; Huang, J.; Tao, J., The prevalence of mild cognitive impairment in type 2 diabetes mellitus patients: a systematic review and meta-analysis. *Acta Diabetol* **2021,** *58* (6), 671-685.

7. Koekkoek, P. S.; Kappelle, L. J.; van den Berg, E.; Rutten, G. E.; Biessels, G. J., Cognitive function in patients with diabetes mellitus: guidance for daily care. *Lancet Neurol* **2015,** *14* (3), 329-40.

8. Kirwan, J. P.; Sacks, J.; Nieuwoudt, S., The essential role of exercise in the management of type 2 diabetes. *Cleve Clin J Med* **2017,** *84* (7 Suppl 1), S15-s21.

9. Wayne, P. M.; Walsh, J. N.; Taylor-Piliae, R. E.; Wells, R. E.; Papp, K. V.; Donovan, N. J.; Yeh, G. Y., Effect of tai chi on cognitive performance in older adults: systematic review and meta-analysis. *J Am Geriatr Soc* **2014,** *62* (1), 25-39.

10. Lin, R.; Cui, S.; Yang, J.; Yang, H.; Feng, Z.; Wahner-Roedler, D. L.; Zhou, X.; Salinas, M.; Mallory, M. J.; Do, A.; Bublitz, S. E.; Chon, T. Y.; Tang, C.; Bauer, B. A.; Xu, M., Effects of Tai Chi on Patients with Mild Cognitive Impairment: A Systematic Review and Meta-analysis of Randomized Controlled Trials. *Biomed Res Int* **2021,** *2021*, 5530149.

11. Guo, S.; Xu, Y.; Qin, J.; Chen, Y.; You, Y.; Tao, J.; Liu, Z.; Huang, J., Effect of tai chi on glycaemic control, lipid metabolism and body composition in adults with type 2 diabetes: A meta-analysis and systematic review. *J Rehabil Med* **2021,** *53* (3), jrm00165.

12. 2. Classification and Diagnosis of Diabetes: Standards of Medical Care in Diabetes-2018. *Diabetes Care* **2018,** *41* (Suppl 1), S13-s27.

13. Alberti, K. G.; Zimmet, P. Z., Definition, diagnosis and classification of diabetes mellitus and its complications. Part 1: diagnosis and classification of diabetes mellitus provisional report of a WHO consultation. *Diabet Med* **1998,** *15* (7), 539-53.

14. Petersen, R. C., Mild cognitive impairment as a diagnostic entity. *J Intern Med* **2004,** *256* (3), 183-94.

15. Nascimento, C. M.; Pereira, J. R.; Pires de Andrade, L.; Garuffi, M.; Ayan, C.; Kerr, D. S.; Talib, L. L.; Cominetti, M. R.; Stella, F., Physical exercise improves peripheral BDNF levels and cognitive functions in mild cognitive impairment elderly with different bdnf Val66Met genotypes. *J Alzheimers Dis* **2015,** *43* (1), 81-91.

16. Fang, Y.; Tao, Q.; Zhou, X.; Chen, S.; Huang, J.; Jiang, Y.; Wu, Y.; Chen, L.; Tao, J.; Chan, C. C., Patient and Family Member Factors Influencing Outcomes of Poststroke Inpatient Rehabilitation. *Arch Phys Med Rehabil* **2017,** *98* (2), 249-255.e2.

17. Elwood, R. W., The Wechsler Memory Scale-Revised: psychometric characteristics and clinical application. *Neuropsychol Rev* **1991,** *2* (2), 179-201.

18. Jaeger, J., Digit Symbol Substitution Test: The Case for Sensitivity Over Specificity in Neuropsychological Testing. *J Clin Psychopharmacol* **2018,** *38* (5), 513-519.

19. RM, R., Validity of the Trail Making Test as an indicator of organic brain damage. *Percept Mot Skills* **1958**.

20. Lucas, J. A.; Ivnik, R. J.; Smith, G. E.; Ferman, T. J.; Willis, F. B.; Petersen, R. C.; Graff-Radford, N. R., Mayo's Older African Americans Normative Studies: norms for Boston Naming Test, Controlled Oral Word Association, Category Fluency, Animal Naming, Token Test, WRAT-3 Reading, Trail Making Test, Stroop Test, and Judgment of Line Orientation. *Clin Neuropsychol* **2005,** *19* (2), 243-69.

21. Becker, J. T.; Boller, F.; Saxton, J.; McGonigle-Gibson, K. L., Normal Rates of Forgetting of Verbal and Non-Verbal Material in Alzheimer's Disease. *Cortex* **1987,** *23* (1), 59-72.

22. Matthews, D. R.; Hosker, J. P.; Rudenski, A. S.; Naylor, B. A.; Treacher, D. F.; Turner, R. C., Homeostasis model assessment: insulin resistance and beta-cell function from fasting plasma glucose and insulin concentrations in man. *Diabetologia* **1985,** *28* (7), 412-9.

23. Sabbatinelli, J.; Castiglione, S.; Macri, F.; Giuliani, A.; Ramini, D.; Vinci, M. C.; Tortato, E.;

Bonfigli, A. R.; Olivieri, F.; Raucci, A., Circulating levels of AGEs and soluble RAGE isoforms are associated with all-cause mortality and development of cardiovascular complications in type 2 diabetes: a retrospective cohort study. *Cardiovasc Diabetol* **2022,** *21* (1), 95.

Statistical Analysis Plan

Effects of Tai chi Chuan on cognitive function in Adults 60 years old or older with type 2 diabetes and mild cognitive impairment: A Randomized Clinical trial

Contents

1 Introduction
2 Methods
  2.1 Funding and Ethical Approval
  2.2 Study design
  2.3 Randomization and Blinding
3 Study outcome variables
  3.1 Primary outcome variable
  3.2 Secondary outcome variables
4 General considerations
  4.1 Population
  4.2 Covariates and subgroup analysis
  4.3 Missing data
  4.4 Interim analysis
  4.5 multi-center difference
5 Statistical analysis
  5.1 Data management and general analysis
  5.2 Analysis of primary endpoint
  5.3 Analysis of other endpoints
  5.4 Sensitivity analysis
  5.5 Software and significant level of the statistical analyses

1 Introduction

The patients with type 2 diabetes mellitus (T2DM) is expected to rise to 439 million in 2030, accounting for 7.7% the population in the world. There are nearly 10 million T2DM patients with mild cognitive impairment (MCI) among people over 65 years old, accounting for about 8% of the people over 65 years old in China. The medical cost for T2DM patients with MCI is 2.5 to 4 times higher than those without T2DM. And T2DM will increase the risk of cognitive impairment, and lead to various complications which will bring serious social and medical economic burden.

It is critically important to identify effective treatments to enhance functional status of T2DM patients with MCI. Exercise has been shown to be beneficial for both type 2 diabetes and mild cognitive dysfunction patients. As a Chinese traditional mind-body exercise that consists of both physical and mediation components, Tai Chi Chuan has been proved to be helpful in global cognition, memory, executive function and attention of MCI, and blood sugar of T2DM. However, the evidence of the effect of Tai Chi Chuan on T2DM patients with MCI is limited. The purpose of this study is to explore the effect of Tai Chi Chuan treating T2DM patients with MCI in cognitive function, blood glucose and biochemistry profile.

2 Methods

2.1 Ethical Approval

This study will be conducted by 4 clinical research centers in China. The study was registered with https://clinicaltrials.gov/. (Register Number: NCT04416841), and approved by the Ethics Committee of (1) The Second People's Hospital Affiliated to Fujian University of Traditional Chinese Medicine, Fuzhou, Fujian, China (approval No. 2020KY-004-02), (2) The Second People's Hospital Affiliated to Heilongjiang University of Traditional Chinese Medicine (approval No.2020-K24), (3) Xiyuan Hospital of China Academy of Chinese Medical Sciences, Beijing, China (approval No. 2020XLA033-2), and (4) Shenzhen Bao'an District People's Hospital, Shenzhen, China (approval No.BYL20200409). All participants provided written informed consent. Furthermore, a Data Security and Monitoring Committee has been established.

2.2 Study design

This is a multi-center, randomized controlled, parallel-group study. The patients will be randomly divided in 1:1:1 ratio into Tai Chi Chuan group, fitness walking group and control group. This study aims to explore the effect of Tai Chi Chuan treating T2DM patients with MCI in cognitive function, blood glucose and biochemistry profile.

2.3 Randomization and Blinding

After signing the informed consent forms, the patients will be enrolled according to the inclusion and exclusion criteria. The patients who meet the eligibility criteria will be randomized into three groups. A statistician unrelated to the trial randomly allocated participants after their enrolment using a secure, central web-based randomization system. The random sequence was generated by SAS version 9.4

(SAS Institute Inc) with a randomized block size of six and stratified by center. The allocation concealment was also conducted by the Research Electronic Data Capture (REDCap).

3 Study outcome variables

3.1 Primary outcome variable

Global cognition-Montreal Cognitive Assessment (MoCA) at 36 weeks

3.2 Secondary outcome variables

1) Global cognition-Montreal Cognitive Assessment (MoCA) at 24 weeks

2) Memory function-Wechsler memory scale at 24 , 36 weeks

3) Digital Symbol test at 24, 36 weeks

4) Trial Making Test part B at 24, 36 weeks

5) Boston naming test at 24, 36 weeks

6) Rey-Osterrieth complex graphics test at 24, 36 weeks

7) Blood glucose metabolism profiles at 24, 36 weeks: fasting blood glucose, glycated hemoglobin (HbA1c), homeostasis model assessment of insulin resistance (HOMA-IR), advanced glycation end products/soluble receptor of advanced glycation end products (AGE/sRAGE).

4 General considerations

4.1 Population

4.1.1 Sample size

To drive all the primary hypothesis tests, the sample size was estimated by using the PASS 15.0 software. Based on a previous study[1] and a pilot study we did. sample size required for each individual hypothesis testing was estimated at the two-sided α level 0.05. With other assumptions, the estimated sample sizes were summarized in **table 1**. Comparisons between Tai Chi Chuan and fitness walking were not made due to lack of trial data. To ensure adequate power for each individual hypothesis testing, at least 87 samples were required for each group, which was 109 cases per group considering 20% dropout rate. Therefore, the total sample size needed for this study was 327 cases.

Table 1. Sample sizes required for each individual hypothesis testing

**Endpoint**	**Comparison**	**Assumptions**	**Sample Size**
primary Endpoint (MOCA)	Tai Chi Chuan vs. Control	●Two sided α = 0.05<br>●80% power<br>●1:1 randomization ratio<br>●Superiority design<br>●Means_{TCC} = 24.24<br>$\text{Means}_{Control}$ = 22.44<br>●$\text{Standard deviation}_{TCC}$ = 2.677<br>$\text{Standard deviation}_{Control}$ = 2.734	SS_{TCC} = 37<br>$SS_{Control}$ = 37
	Physical exercise vs. Control	●Two sided α = 0.05<br>● 80% power<br>●1:1 randomization ratio<br>●Superiority design<br>●Means_{PE} = 23.90<br>$\text{Means}_{Control}$ = 22.08<br>●$\text{Standard deviation}_{PE}$ =3.5<br>$\text{Standard deviation}_{Control}$ =4.9	SS_{FT} = 87<br>$SS_{Control}$ = 87
SS = Sample Size; TCC = Tai Chi Chuan; PE= Physical exercise; FT = Fitness walking			

4.1.2 Data set

Intention-to-treat (ITT) refers to all eligible patients who were randomized to treatment, include drop-out cases. Multiple imputation methods were used for missing observations.

Modified intention-to-treat (mITT) refers to all randomized subjects who receive at least 12 weeks intervention. Primary analyses were conducted using Primary outcome are analyzed according to the multiple imputation methods for missing observations. Missing values for secondary efficacy outcomes are analyzed according to the actual data obtained.

Per protocol(PP) refers to the set of cases that meet the inclusion criteria, do not meet the exclusion criteria, and have completed the treatment protocol. The PPS is a subset of the FAS in which each subject in the dataset is a valid case or sample with good adherence, no protocol violations, and complete baseline values for key indicators. Prespecified sensitivity analyses were conducted using complete-case data.

Safety set (SS) refers to the actual data for subjects who receive at least one intervention after randomization and for whom safety indicators are documented. The incidence of adverse reactions is calculated using the number of cases in the safety set.

Adverse events: Will be analyzed, with the number of cases, category, and severity of occurrences counted separately and their relationship to the intervention.

4.2 Covariates and subgroup analysis

As this is an RCT study, the probability of imbalance in baseline data between the three groups is speculated to be 5% (a minor probability event). Therefore, no multivariate analysis with the adjustment of covariates will be performed to analyze primary endpoint. In addition, subgroup analysis will be performed by sex, age, education, BMI at baseline, disease duration, HbA1c at baseline, comorbidity, site. The subgroup analysis will be carried out as exploratory.

4.3 Missing data

Due to the long duration of intervention and the long follow-up period, so there may be a lot of missing data. If missing data were found, the percentage of missing data will be reported, the potential patterns of missing data should be examined, and appropriate method should be used for multiple imputation of missing data. The multiple imputation method will be preferred for analyzing the missing data, and the complete-case data should be reported in the manuscript as sensitivity analysis. The patients' demographic characteristics were involved in the missing data model for multiple imputation, and the number of multiple imputation will be set as 10.

4.4 Interim analysis

No interim analysis is planned in this study.

4.5 multi-center effect analysis

This is a multicenter RCT study, and there may be central effects among different centers. The subgroup analysis will be used to compare the multi-center effect.

5 Statistical analysis

5.1 Data management and general analysis

Research Electronic Data Capture (REDCap) dataset system will be used for data collection and management. Independent data management committee and data monitoring board is responsible for the management of validity and effectiveness of the data.

The data analyses mainly include statistical description and statistical inference. Quantitative data will be described by central tendency and dispersion tendency. The normally distributed data of central tendency and dispersion tendency will be described as means and standard division, respectively. The non-normally distributed data of central tendency and dispersion tendency will be described as median and quartiles. The qualitative data will be described as frequency and percentage. Statistical inference, independent t test or non-parameter test will be used to compare the quantitative data between the three groups, while chi-square test or Fisher's exact test will be used for comparing the qualitative data between the three groups.

5.2 Analysis of primary endpoint

The Type I error rate (α-level) used in the assessment of pair-wise treatment comparisons for the primary efficacy endpoints is 5%.

The assessment of significance for the exercise interventions versus control group contrasts will use a step-down testing strategy within the primary efficacy endpoint with the multiple imputation approach (ITT, mITT and PP data set). The step-down testing will first test Tai Chi Chuan versus control, and if statistically significant($p \leq 0.05$) will then test Tai Chi Chuan versus fitness walking. We also compared treatment effects in each group after adjusting for patient self-reported dietary calories and physical activity on the basis of a generalized linear model. As expected, the adjusted and unadjusted findings were similar.

5.3 Analysis of secondary endpoints

For secondary and exploratory endpoints, continuous data will be presented as means (SDs) or median (IQRs), as appropriate. The secondary endpoint followed no Gaussian distribution, will be presented as median (interquartile range) and tested by analysis of variance (ANOVA). Because of the potential for type I error due to multiple comparisons, findings for the analyses of the secondary outcomes should be interpreted as exploratory

5.4 Sensitivity analysis

Prespecified sensitivity analyses were conducted using complete-case data. Prespecified sensitivity analyses were conducted for all participants using multiple imputation methods for missing observations based on baseline and, when available, post-intervention and follow-up data assuming missing data are missing at random.

5.5 Software and significant level of the statistical analyses

All statistical analyses were conducted using SPSS, 24.0 (IBM, Armonk, NY, USA) and R, version 4.2.1 software (The R Project for Statistical Computing, www.r-project.org). Statistical significance was defined as $P < .05$ with 2-sided testing, except for pairwise multiple comparisons for which the significance level was adjusted by the least significant difference (LSD) correction.

1. Nascimento, C. M.; Pereira, J. R.; Pires de Andrade, L.; Garuffi, M.; Ayan, C.;

Kerr, D. S.; Talib, L. L.; Cominetti, M. R.; Stella, F., Physical exercise improves peripheral BDNF levels and cognitive functions in mild cognitive impairment elderly with different bdnf Val66Met genotypes. *J Alzheimers Dis* **2015**, *43* (1), 81-91.
