# Supplementary material for: Effects of Tai Chi Chuan on Cognitive Function in Adults 60 Years or Older With Type 2 Diabetes and Mild Cognitive Impairment in China: A Randomized Clinical Trial
Source: JAMA Netw Open. 2023 Apr 6;6(4):e237004. doi: 10.1001/jamanetworkopen.2023.7004 (PMC10080376; doi:10.1001/jamanetworkopen.2023.7004)
Supplement: Supplement 2. — eTable 1. Medians (IQR) of Diabetes Medication, Patient Self-reported Dietary Calories, and Physical Activity of 3 Groups by ITT eTable 2. Mean Difference (SD) of MoCA in 3 Groups by MITT eTable 3. Generalized Linear Model of MoCA and Physical Activity by ITT eTable 4. Safety Outcomes: Adverse Events eFigure. Subgroup Analysis of MoCA in 24 Weeks by ITT [file jamanetwopen-e237004-s002.pdf]

Supplementary Online Content

Chen Y, Qin J, Tao L, et al. Effects of tai chi chuan on cognitive function in adults 60 years or older with type 2 diabetes and mild cognitive impairment in China: a randomized clinical trial. *JAMA Netw Open*. 2023;6(4):e237004. doi:10.1001/jamanetworkopen.2023.7004

eTable 1. Medians (IQR) of Diabetes Medication, Patient Self-reported Dietary Calories, and Physical Activity of 3 Groups by ITT
eTable 2. Mean Difference (SD) of MoCA in 3 Groups by MITT
eTable 3. Generalized Linear Model of MoCA and Physical Activity by ITT
eTable 4. Safety Outcomes: Adverse Events
eFigure. Subgroup Analysis of MoCA in 24 Weeks by ITT

This supplementary material has been provided by the authors to give readers additional information about their work.

© 2023 Chen Y et al. *JAMA Network Open*.

eTable 1. Medians (IQR) of Diabetes Medication, Patient Self-reported Dietary Calories, and Physical Activity of 3 Groups by ITT

ITT	Median(IQR) Tai Chi Chuan (n=107)	Fitness Walking (n=110)	Control (n=111)
**Diabetes medication**			
baseline	2(1)	2(1)	2(1)
24weeks	2(1)	2(1)	2(1)
36weeks	2(1)	2(1)	2(1)
**Dietary[a] (calorie)**			
baseline	1163(549)	1086(677)	1186(758)
24weeks	1038(521)	1049(534)	1135(744)
36weeks	1214(435)	1215(366)	1274(596)
**Total METs[b] ($min.wk^{-1}$)**			
baseline	1188(1287)	1270(1346)	1095(1487)
24weeks	2011(1416)	2187(1678)	2290(2038)
36weeks	1540(1000)	1350(864)	1379(969)
**Vigorous METs[c] ($min.wk^{-1}$)**			
baseline	33(116)	43(139)	110(191)
24weeks	147(208)	125(191)	193(237)
36weeks	56(82)	53(121)	104(336)
**Moderate METs [d] ($min.wk^{-1}$)**			
baseline	514(542)	439(521)	296(537)
24weeks	968(745)	1005(877)	1033(645)
36weeks	894(735)	738(522)	625(568)
**Walking METs[e] ($min.wk^{-1}$)**			
baseline	641(964)	788(1088)	689(1079)
24weeks	1383(641)	1505(809)	1471(744)
36weeks	610(610)	573(469)	691(583)
**Sedentary time (min)**			
baseline	305(138)	304(146)	280(150)
24weeks	309(142)	299(139)	301(119)
36weeks	355(135)	340(109)	327(125)

[a] Dietary assessment using Self-Reported 24-Hour Recalls.

[b]METs=Amount of metabolic equivalent minutes per week （METs $min.wk^{-1}$） in the IPAQ-S categories.

[c]Vigorous intensity =8.0 METs

[d]Moderate intensity =4.0 METs

[e]Walking intensity =3.3 METs

© 2023 Chen Y et al. *JAMA Network Open*.

eTable 2. Mean Difference (SD) of MoCA in 3 Groups by MITT

	Tai Chi Chuan	Fitness Walking	Control	*P* value	Between-group change, mean (95% CI)		
					Tai Chi Chuan vs Fitness Walking	Tai Chi Chuan vs control	Fitness Walking vs Control
MITT, n	101	103	99				
MoCA, mean(SD), (range 0-30)							
baseline	21.38(2.76)	21.51(2.51)	21.29(2.8)	.84	-0.14(-0.88 to 0.6)	0.08(-0.67 to 0.83)	0.22(-0.52 to 0.97)
24 weeks	24.08(3.12)	23.6(3.43)	22.48(3.45)	.003	0.48(-0.44 to 1.40)	1.59(0.67 to 2.52) *	1.12(0.19 to 2.04) *
36 weeks	24.81(2.65)	23.92(3.24)	22.69(3.34)	< .001	0.89(0.04 to 1.74) *	2.13(1.27 to 2.98) *	1.24(0.38 to 2.09) *

© 2023 Chen Y et al. *JAMA Network Open*.

eTable 3. Generalized Linear Model of MoCA and Physical Activity by ITT

ITT		β	SE	*P*
24weeks_MoCA	Tai Chi Chuan	0.000		
	Fitness Walking	-0.559	0.3654	.126
	Control	-1.618	0.3722	＜.001
36weeks_MoCA	Tai Chi Chuan	0.000		
	Fitness Walking	-0.935	0.355	.009
	Control	-2.031	0.362	＜.001

Note: Adjusted after T0_MoCA and T0_vigorous METs

eTable 4. Safety Outcomes: Adverse Events

	Tai Chi Chuan (n=107)	Fitness Walking (n=110)	Control (n=111)	*P*
Adverse events		Participants with events, No.		
Total adverse events	8	13	16	.263
Hypoglycemia	1	2	0	
Fall	5	10	15	.077
Fracture	1	1	0	
Hospitalizations	0	0	1	
Emergency department visits	1	0	0	

© 2023 Chen Y et al. *JAMA Network Open*.

eFigure. Subgroup Analysis of MoCA in 24 Weeks by ITT

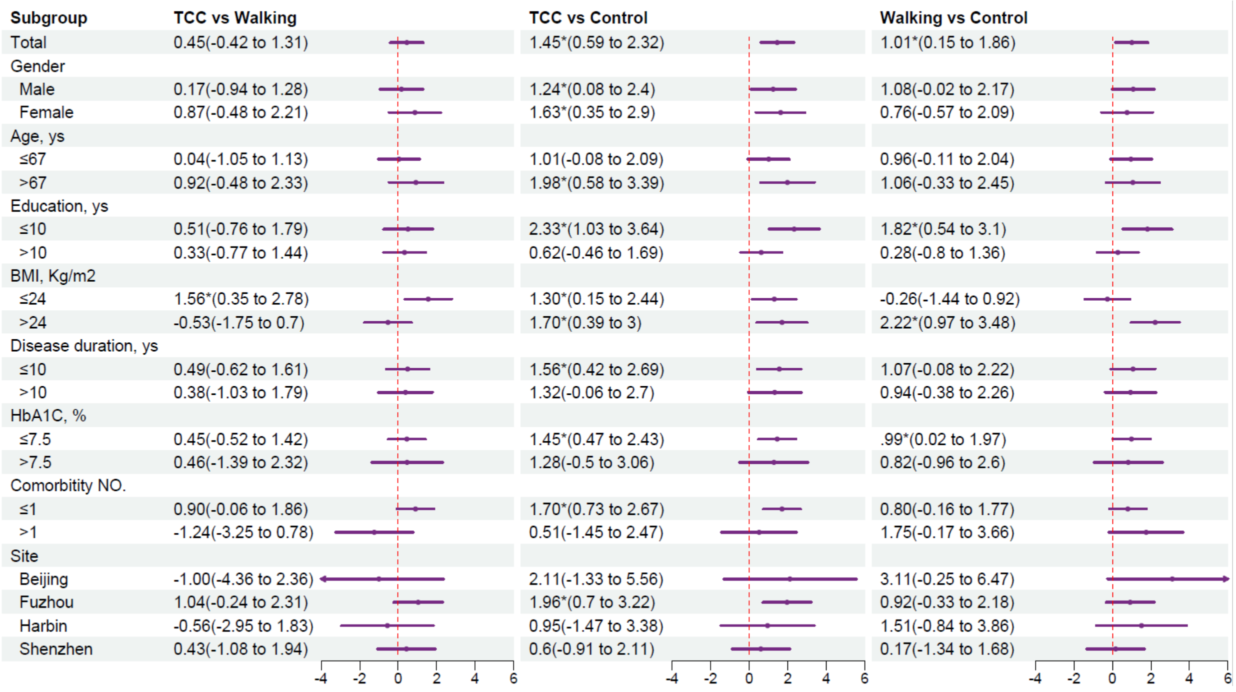

© 2023 Chen Y et al. *JAMA Network Open*.
