# Supplementary material for: Effects of Tai Chi Chuan on Cognitive Function in Adults 60 Years or Older With Type 2 Diabetes and Mild Cognitive Impairment in China: A Randomized Clinical Trial
Source: JAMA Netw Open. 2023 Apr 6;6(4):e237004. doi: 10.1001/jamanetworkopen.2023.7004 (PMC10080376; doi:10.1001/jamanetworkopen.2023.7004)
Supplement: Supplement 3. — Data Sharing Statement [file jamanetwopen-e237004-s003.pdf]

Data Sharing Statement

Chen. Effects of Tai Chi Chuan on Cognitive Function in Adults 60 Years or Older With Type 2 Diabetes and Mild Cognitive Impairment in China. *JAMA Netw Open*. Published April 06, 2023. doi:10.1001/jamanetworkopen.2023.7004

Data
Data available: Yes
Data types: Deidentified participant data
How to access data: How to access data: Send request email to Professor Tao, taojing01@fjtcm.edu.cn
When available: With publication

Supporting Documents
Document types: None

Additional Information
Who can access the data: anyone requesting the data
Types of analyses: for any purpose
Mechanisms of data availability: with investigator support
